# Pontine tegmental cap dysplasia: developmental and cognitive outcome in three adolescent patients

**DOI:** 10.1186/1750-1172-6-36

**Published:** 2011-06-08

**Authors:** Marilena Briguglio, Lorenzo Pinelli, Lucio Giordano, Alessandro Ferraris, Eva Germanò, Serena Micheletti, Mariasavina Severino, Laura Bernardini, Sara Loddo, Gaetano Tortorella, Francesca Ormitti, Roberto Gasparotti, Andrea Rossi, Enza Maria Valente

**Affiliations:** 1 Division of Child Neurology and Psychiatry, Dept. of Medical and Surgical Pediatric Sciences, University of Messina, Messina, Italy; 2 Dept. of Neuroradiology, Spedali Civili di Brescia, Brescia, Italy; 3 Division of Child Neurology, Spedali Civili di Brescia, Brescia, Italy; 4 IRCCS Casa Sollievo della Sofferenza, Mendel Laboratory, San Giovanni Rotondo (FG), Italy; 5 Dept. of Neuroradiology, IRCCS G. Gaslini Children's Hospital, Genoa, Italy; 6 Dept. of Neuroradiology, University of Parma, Parma, Italy; 7 Unit of Genetics and Pediatric Immunology, Dept. of Medical and Surgical Pediatric Sciences, University of Messina, Messina, Italy

## Abstract

Pontine Tegmental Cap Dysplasia (PTCD) is a recently described, rare disorder characterized by a peculiar cerebellar and brainstem malformation. Nineteen patients have been reported to date, of which only one in the adolescent age, and data on the clinical, cognitive and behavioural outcome of this syndrome are scarce.

Here we describe three adolescent patients with PTCD. All presented bilateral deafness and multiple cranial neuropathies, variably associated with skeletal, cardiac and gastro-intestinal malformations. Feeding and swallowing difficulties, that are often causative of recurrent aspiration pneumonias and death in the first years of life, completely resolved with age in all three patients. Neuropsychological assessment showed borderline to moderate cognitive impairment, with delay in adaptive functioning, visual-spatial and language deficits. Two of three patients also showed mild behavioural problems, although their overall socialization abilities were well preserved. Cochlear implantation in two patients significantly improved their relational and learning abilities. Fibre tractography confirmed the abnormal bundle of transversely oriented fibres forming the typical pontine "tegmental cap" and absence of decussation of the superior cerebellar peduncles, supporting the hypothesis that PTCD results from abnormal axonal guidance and/or migration.

These data indicate that PTCD may have a favourable long-term outcome, with borderline cognitive deficit or even normal cognition and partially preserved speech.

## Background

Pontine Tegmental Cap Dysplasia (PTCD) is a recently described syndrome that was reported to date only in 19 patients [1-9]. The diagnostic signature of PTCD stems from a peculiar constellation of hindbrain malformations, including cerebellar vermis hypo-dysplasia, absence of inferior olives and near absence of middle cerebellar peduncles, lateralized superior cerebellar peduncles with shortening of the isthmus, flattened ventral pons, and vaulted pontine tegmentum (the so-called "tegmental cap"). Fibre tractography performed in two patients showed an abnormal transverse bundle of fibres in the upper pons forming the cap [1,2].

Patients typically present with neonatal hypotonia, pyramidal and cerebellar signs, multiple deficits of the cranial nerves and extracranial malformations comprising cardiac, gastrointestinal, genitourinary and skeletal defects, especially of the vertebrae and ribs. The cranial neuropathies result in the variable association of neurosensorial deafness, abnormal eye movements, visual reduction due to corneal clouding, facial paralysis, and difficulties in chewing and swallowing that may require positioning of nasogastric tube or gastrostomy.

Severe developmental delay has been reported in most cases, with bilateral deafness concurring to the absence of language or severe speech disorder. However, actual data on the spectrum of cognitive impairment in these patients are scarce, and a detailed neuropsychological assessment has never been performed, especially in the long term. Indeed, only one out of 16 clinically described patients had reached adolescent age at latest examination, and some case reports are mostly focused on the neuroimaging data, with limited clinical information.

Here we present three Italian adolescent patients with PTCD, show tractography data for two of them, and report a detailed characterization of their neuropsychological profile with respect to the different cognitive and behavioural areas.

## Case Presentation

### Case reports

All patients were born of non consanguineous parents with negative family history and uneventful pregnancies.

#### Patient 1

This 14 year old boy presented with microcephaly at birth (< 2SD), hypotonia and delayed milestones. The clinical picture was characterized by bilateral severe hearing impairment, decreased corneal sensation, ptosis, horizontal nystagmus and limited vertical pursuit, and chewing and swallowing difficulties that progressively improved with age, allowing him a normal solid diet. At three years, he acquired the ability to walk unsupported for a few steps. Neurological examination showed truncal and gait ataxia, bilateral ankle clonus with hyperreflexia and Babinski sign. From age 8 years, he developed atypical absences and tonic-clonic seizures that were well controlled by Valproate and Carbamazepine treatment. The only extracranial anomaly in this patient was the presence of atrial septal defect associated with long QT interval.

#### Patient 2

This 13 year old girl has been previously reported as the first PTCD patient undergoing cochlear implantation at age 11 [8]. She was hypotonic at birth and developmental milestones were delayed. There were multiple cranial neuropathies that caused bilateral deafness, corneal anaesthesia, left facial paralysis, strabismus and vertical nystagmus, chewing and swallowing difficulties that progressively resolved with age. Clinical examination disclosed pyramidal signs such as brisk tendon reflexes and ankle clonus, as well as truncal, gait and limb ataxia, but the patient was able to walk unsupported for a few steps. Extracranial anomalies comprised esophageal atresia with tracheoesophageal fistula that was corrected at birth, ventricular septal defect, multiple thoracic hemivertebrae, and partial fusion of ribs 4-5 on the right side. Before implantation (with hearing aids), she demonstrated no detection of speech and had unintelligible connected speech; at the last follow-up (22 months post-implantation) she reached the word identification level and had intelligible connected speech.

#### Patient 3

This 15 year old girl presented with hypotonia, developmental delay, bilateral deafness, decreased corneal sensation, horizontal nystagmus, strabismus, and feeding problems due to chewing and swallowing difficulties. Similarly to the other two patients, these progressively resolved with age. There were deep tendon hyperreflexia, truncal ataxia and dysmetria. Extracranial anomalies included atrial septal defect, scoliosis and split of a cervical vertebra. She also underwent cochlear implantation at age 13, with mild improvement of hearing but reported significant progress in her relational and learning abilities.

### Clinical and instrumental testing

#### Imaging studies

**Figure 1:**
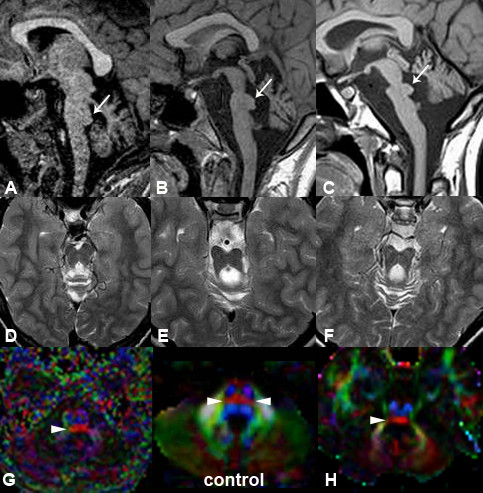
**Neuroimaging of PTCD patients**. MRI and DTI at 1.5 T of patient 1 (A, D, G), patient 2 (B, E) and patient 3 (C, F, H). In all the patients, sagittal T1-weighted images (A, B, C) show the typical "cap" (arrows) in the pontine tegmentum protruding into the fourth ventricle, diagnostic for the malformation. Note also the flat ventral pons and dysplastic cerebellar vermis. T2-weighted images (D, E, F) at the level of the isthmus reveal a "molar tooth-like" appearance, with laterally displaced superior cerebellar peduncles. Colour-coded fractional anisotropy maps of patient 1 (G) and patient 3 (H) are shown with standard DTI conventions (blue for superior↔inferior direction perpendicular to plane of section, red for right↔left direction, green for anterior↔posterior direction). The tegmental "cap" (arrowhead) is a bundle of transversely oriented fibres, possibly representing "ectopic" pontine transverse fibres, normally located in the midpons (double arrowheads in "control").

In all three patients, brain MRI showed a hypoplastic pons with flattened ventral profile, a "shrunken" cerebellum with dysplastic vermis, and the typical focal bulging of the pontine tegmentum projecting into the fourth ventricle ("tegmental cap"), that is diagnostic for the malformation. Additional findings, frequently encountered in PTCD, included absence of the normal lateral bulging of the medulla (possibly due to absence of the inferior olivary nucleus), thinning of the ponto-mesencephalic junction, bilateral abnormalities of all the cerebellar peduncles (hypoplastic or absent the inferior and the middle ones, laterally displaced the superior ones with a "molar tooth-like" appearance). In all three patients, the corpus callosum and hippocampal formation were fully developed and the anterior commissure was visible; there were no cortical malformations nor white matter signal abnormalities. In patient 2, frontal sulci were slightly enlarged. Bilateral stenosis of the internal auditory canals with severely hypoplastic/absent cochlear nerve was a constant finding; in all three patients the inner ear was normal (Figure 1).

**Figure 2:**
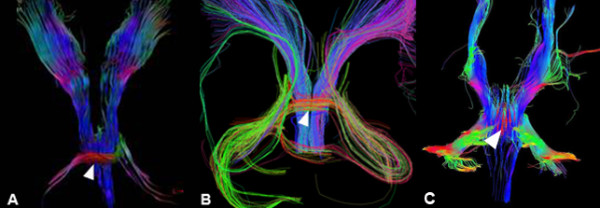
**Tractography study**. 3D projection of brainstem tractography (posterior view) at 1.5 T in patient 1 (A), patient 3 (B) and a normal control (C) with 16, 12 and 30 non-collinear diffusion-encoding directions, respectively. In both patients (A, B) the red streamlines of the pontine tegmentum "cap" are clearly visible (arrowhead): some of them continue into the expected location of the middle cerebellar peduncles, possibly representing fibres projecting to the cerebellum. In the normal subject (C), red streamlines (arrowhead) represent the more anteriorly located pontine trasverse fibers, partially "covered" by fibers running longitudinally in the brainstem tegmentum.

Diffusion tensor imaging (DTI) with color-coded fractional anisotropy (FA) maps and tractography were obtained at 1.5 T in patients 1 and 3, with 16 and 12 non-collinear diffusion-encoding directions, respectively. In both patients, FA maps showed that the pontine "tegmental cap" is a bundle of transversely oriented fibres, possibly representing ectopic fibres normally located in the midpons and connecting the cerebellar hemispheres via the middle cerebellar peduncles. The mesencephalic decussation of the superior cerebellar peduncles was not visible (Figures 1, 2).

#### Neuropsychological assessment

Cognitive assessment was performed using the non-verbal Scale Leiter-R. Despite the expressive and perceptive deficits related to the severe hearing loss in all patients, cognitive impairment ranged from mild-moderate mental retardation with better performances in visuo-spatial tasks (patients 1 and 2) to borderline full scale IQ (patient 3).

Neurolinguistic assessment in patients 1 and 2 showed moderate deficit of expressive and receptive language, characterized by phonological, lexical and morphosyntactic difficulties in both production and comprehension. In particular, both patients were able to produce single-word sentences (with understandable bi-trisillabic words) that were used in a functional way; they also spontaneously used several descriptive and conventional gestures. On the comprehension tasks, both patients were deficient in receptive vocabulary (Peabody test: < 65) and in syntactic comprehension of complex sentences. Their receptive level was characterized by verbal comprehension of daily routine words and short sentences (simple orders). In patient 3, expressive language was simple and used for request, but overall less impaired than in the other patients. All cases presented dysarthria and phono-articulatory deficits.

Visuoperceptual and graphic tasks were also abnormal, with reading more impaired than writing. Patients 2 and 3 could write simple words by dictation and read a simple text. Patient 1 showed a greater delay in graphic and learning abilities, being able to write only 2-3 simple words and read simple syllables only.

Adaptive functioning assessed with the Vineland Adaptive Behaviour Scales (VABS) was significantly delayed in patients 1 and 2, who showed poor daily living skills, with an equivalent age of 4 years. Daily self-care activities such as personal hygiene, dressing and undressing, eating, and voluntarily controlling urinary and fecal discharge were possible with assistance. Patient 3 performed better, with daily living skills equivalent to an age of 8 years.

Finally, the behavioural profile was assessed with the CBCL scale. Mild behavioural problems were observed especially in patients 1 and 2, including emotional fragility, attention problems, poor tolerance to frustration (oppositional behaviour related to environmental stress), and unstable mood. However, none of these problems was consistent with a psychiatric diagnosis.

#### Genetic testing

After obtaining a normal standard karyotype, all patients underwent high resolution SNP-array analysis to search for genomic imbalances that could be related to the disease pathogenesis. This was performed on genomic DNA extracted from peripheral blood, on a GeneChip SNP-array 6.0 Platform (Affymetrix, Santa Clara, CA), that includes approximately 900,000 SNPs and 900,000 copy-number probes at an average distance of 0.7 Kb. The procedures for DNA digestion, fragmentation, labelling and hybridization were performed as previously reported [10]. Data analysis was performed by Genotyping Console 4.0, with a default setting of at least 25 markers showing consensus for gain or loss spanning at least a 75 Kb region. With these settings, no pathogenic copy number variants were identified in any of the three patients.

## Conclusions

Pontine Tegmental Cap Dysplasia is a novel syndrome that can be easily diagnosed upon recognition of its peculiar clinical and neuroradiological features. An early diagnosis is critical to plan a complete diagnostic workup and clinical follow-up, that should include neurological, audiological and ophthalmological assessment, a skeletal X-ray, and the search for possibly associated gastrointestinal, genitourinary and cardiac defects.

The prognosis of PTCD-related phenotype appears to be highly variable. Four patients died in the first two years of life due to early complications such as recurrent aspiration pneumonias. However, it must be said that chewing and swallowing problems tend to significantly improve with age, and life expectancy considerably increases once patients have survived infancy. Indeed, of 18 patients for whom clinical details are available, five were in the second decade of life, and none of them presented any life-threatening active complication [2-3, present study].

The neuropsychological outcome of PTCD patients is also variable, ranging from severe mental retardation up to normal cognition (IQ = 94) in a 7 year old child [1]. Our report is the first to present a detailed cognitive and behavioural assessment of PTCD patients who have reached adolescent age. Cognitive impairment in our three patients ranged from mild-moderate mental retardation to borderline IQ, with delay in adaptive functioning, visual-spatial and language deficits. Two of three patients also showed behavioural problems including mild emotional fragility, attention deficit, poor tolerance to frustration and unstable mood, although their overall socialization abilities were well preserved. These findings are in line with previous studies on children and adults with acquired cerebellar lesions or congenital cerebellar malformations, providing substantial evidence pointing to the involvement of the cerebellum in processing higher-order non-motor functions. Indeed, both acquired and congenital cerebellar lesions lead to the development of a complex behavioural pattern termed "Cerebellar Cognitive Affective Syndrome (CCAS)", characterized by cognitive disabilities with disorders of attentional functions, visual-spatial abilities, language and affectivity [11-14].

Language impairment is a constant feature in PTCD, ranging from complete absence of language with or without sign language to a more understandable speech with severe to moderate deficit in both the expressive and receptive areas. This is mainly related to the bilateral profound hearing impairment or sensorineural deafness due to the absence or marked hypoplasia of the VIII cranial nerve. In these cases, the rehabilitative effect of hearing aids is usually weak, and it is expected that cochlear implantation also may not be effective. However, right cochlear implantation in two of our patients resulted in a significant improvement of the intelligibility of speech, increasing their self-confidence and social integration [8].

The pathogenesis of PTCD is still unknown, although it has been postulated that this complex cerebellar and brainstem malformation may derive from a defect in axonal guidance and/or migration [1,2]. PTCD must be distinguished from a far more common group of disorders also characterized by a peculiar mid-hindbrain malformation and presenting at birth with hypotonia and abnormal eye movements, Joubert syndrome and related disorders (JSRD) [15]. The neuroradiological signature of JSRD is represented by the "molar tooth sign" (MTS) which stems from the association of cerebellar vermis hypo-dysplasia, deepened interpeduncular fossa, and thickened, elongated and horizontalized superior cerebellar peduncles. The absence of decussation of the superior cerebellar peduncles seems to be a shared feature in both conditions, and an "MTS-like" appearance of the midbrain has been described in some PTCD patients [1,2,6]. However, in these cases the superior cerebellar peduncles appear laterally displaced but less elongated and horizontalized than in the classical MTS. Moreover, the typical "pontine tegmental cap" is never seen in JSRD. Clinical features are also different, insofar the multiple cranial neuropathies and the vertebral and rib anomalies that are typical of PTCD are never seen in JSRD, which pattern of multiorgan involvement includes retinopathy, progressive nephronophthisis (NPH), congenital hepatic fibrosis and polydactyly.

A correct differential diagnosis is of paramount important not only for clinical follow-up and prognosis of patients, but also to appropriately counsel the families for inheritance. In fact, JSRD are autosomal recessive disorders with a recurrence risk of one in four for each subsequent pregnancy, while the genetic basis of PTCD still remains to be determined. Both sexes are equally affected and all reported patients are sporadic, with negative family history and absence of parental consanguinity. These observations suggest that PTCD may be caused by a *de novo *heterozygous autosomal genetic defect acting in a dominant manner. Recently, one patient has been described who carried a heterozygous 96 Kb deletion on chromosome 2q13 comprising the NPHP1 gene, that the authors considered causative of PTCD [3]. The homozygous deletion of a 250 Kb genomic region encompassing the NPHP1 gene is known to cause a group of ciliopathies including isolated juvenile NPH, Senior-Löken syndrome (NPH associated with retinal dystrophy), and JSRD with NPH. Yet, it is unlikely that a heterozygous NPHP1 deletion may be pathogenically associated to PTCD. In fact, patients homozygous for the NPHP1 deletion usually inherit such genomic imbalance from asymptomatic parents, who are both heterozygous carriers. Moreover, juvenile NPH is a constant feature associated with NPHP1 deletions/mutations, that becomes clinically manifest towards the end of the first or early second decade of life with acute or chronic renal insufficiency [16]. None of the PTCD patients described to date, including the 16 year old patient carrier of the NPHP1 deletion, presented signs of NPH or renal failure. Taken together, these observations suggest that the small chromosome 2q deletion detected in one PTCD patients may not be the genetic cause of this syndrome but just represent a coincidental finding or, possibly, a genetic modifier of the phenotype. In our study, SNP-array analysis at a resolution of 75 Kb excluded the presence of pathogenic genomic imbalances in all three patients, suggesting that point mutations in a single gene or micro-rearrangements may be causative for PTCD.

In conclusion, PTCD is a rare, but likely underestimated syndrome characterized by a peculiar cerebellar and brainstem congenital defect, and a specific constellation of clinical signs. Although the severity of the phenotypic spectrum and the extent of multiorgan involvement is variable, it is important to stress that some patients have a favorable long-term outcome, with borderline cognitive deficit or even normal cognition and partially preserved speech. Further studies on larger cohorts are needed to unravel the underlying genetic cause of this syndrome and to better understand its pathogenetic mechanisms.

## Competing interests

The authors declare that they have no competing interests.

## Authors' contributions

MB, LG, EG and SM performed the clinical evaluation and neuropsychological assessment of patients; LP, FO, MS, RG and AR performed/revised the brain MRIs and carried out the DTI-tractography study; AF, LB and SL performed genetic studies; MB and EMV were involved in the conception of the project and wrote the manuscript; AR, LG, LP and GT participated in the design of the study and revised the paper for intellectual content. All authors read and approved the final manuscript.
